# Thyroid cancer risk in airline cockpit and cabin crew: a meta-analysis

**DOI:** 10.1186/s41199-018-0034-8

**Published:** 2018-08-17

**Authors:** George S. Liu, Austin Cook, Michael Richardson, Daniel Vail, F. Christopher Holsinger, Ingrid Oakley-Girvan

**Affiliations:** 1 Stanford University School of Medicine, Stanford University, 450 Serra Mall, Stanford, CA 94305 USA; 2 Department of Otolaryngology–Head and Neck Surgery, Stanford University, 801 Welch Road, Stanford, CA 94305 USA; 3 Cancer Prevention Institute of California, Fremont, CA 94538 USA; 4 Department of Health Research and Policy, the Canary Center at Stanford for Cancer Early Detection and the Stanford Cancer Institute, Stanford University School of Medicine, Stanford, CA 94305 USA; 5 Public Health Institute, Oakland, CA 94607 USA

**Keywords:** Airline, Pilot, Cabin crew, Thyroid cancer, Incidence, Risk, Meta-analysis

## Abstract

**Background:**

Airline crew are exposed to ionizing radiation as part of their occupation and have a documented increased risk of melanoma and cataracts. However, whether their occupation predisposes them to an increased risk of thyroid cancer is not established. The purpose of this systematic review and meta-analysis was to assess the risk of thyroid cancer in airline cockpit and cabin crew compared with the general population.

**Methods:**

The MEDLINE database accessed via PubMed and Cochrane Database were searched. We included cohort studies reporting the standardized incidence ratio (SIR) or standardized mortality ratio (SMR) of thyroid cancers in any flight-based occupation.

**Results:**

Of the 1777 citations retrieved in PubMed, eight studies with a total of 243,088 aircrew members and over 3,334,114 person-years of follow-up were included in this meta-analysis. No relevant studies were identified on Cochrane Database. The overall summary SIR of participants in any flight-based occupation was 1.11 (95% CI, 0.79–1.57; *p* = 0.613; 6 records). The summary SIR for cockpit crew was 1.21 (95% CI, 0.75–1.95; *p* = 0.383; 4 records) and the summary SIR for cabin crew was 1.00 (95% CI, 0.60–1.66; *p* = 0.646; 2 records). The overall summary standardized mortality ratio for airline crew was 1.19 (95% CI, 0.59–2.39; *p* = 0.773; 2 records).

**Conclusion:**

Airline crew were not found to have a significantly elevated risk of thyroid cancer incidence or mortality relative to the general population. Future research should capitalize on the growing occupational cohort dataset and employ innovative methods to quantify lifetime radiation exposure to further assess thyroid cancer risk in airline crew.

**Electronic supplementary material:**

The online version of this article (10.1186/s41199-018-0034-8) contains supplementary material, which is available to authorized users.

## Background

Commercial airline crew spend hundreds of hours per year working at cruising altitudes around 9–12 km near the tropopause [1]. Flying high reduces drag and turbulence [2] but also reduces atmospheric protection from cosmic rays [3], high-energy radiation from extra-galactic supernovae [4]. With less atmospheric protection during flights, airline crew are exposed to greater doses of radiation. This is estimated to add an additional 2–9 mSv per year [1, 5] to the typical annual exposure from natural background radiation of 2.4 mSv [6]. By comparison, a typical dose from an adult CT scan is about 15 mSv [7].

Airline crews may represent a natural experiment due to work conditions that increase circadian disruption and radiation exposure. Historically several cancers such as melanoma [8] and breast cancer [9–11] have been observed at higher rates in this population. While elevated early stage breast cancer rates might be more related to differences in parity and age of first birth than exposure to cosmic radiation [12], melanoma rates could reflect a true increase related to increased cosmic radiation. However UV radiation exposure may also be a cause [13], and few studies have evaluated other risk factors that could underlie increased melanoma incidence, such as history of sunburn, on-ground sun exposure, and skin type [14].

Radiation poses a theoretical health risk because of its potential to damage DNA and foster mutagenesis [15, 16]. Though the estimated radiation exposure by airline workers is less than the annual occupational exposure limit of 20 mSv recommended by the International Commission on Radiological Protection [17], there is still concern for radiation effects on health given the observed increased incidence of neoplastic disease [8–11], cataracts [18] and miscarriage [19] in this epidemiological cohort. Of course, there are multiple potential confounding factors in assessing these risks including socioeconomic status, increased health surveillance, cigarette smoke exposure prior to the ban of smoking on U.S. flights in 1990, and circadian rhythm disruption [20].

Among organs susceptible to radiation, the thyroid is particularly sensitive. Previous reports have confirmed an increased incidence of thyroid cancer in individuals exposed to high levels of radiation during childhood or adolescence [21–23]. These investigations primarily studied patients exposed to radiation either during diagnostic radioiodine workup [24] or from fallout resulting from high dose nuclear catastrophes, such as the atomic bombings of Hiroshima and Nagasaki [25] and the accident at the Chernobyl nuclear power plant [21, 26]. Despite the known sensitivity of the thyroid to radiation, the increased levels of ionizing radiation sustained by airline crew personnel, and the higher incidences of other cancers in this population, it remains poorly understood whether airline crew members have an increased risk of thyroid cancer.

The incidence of thyroid cancer continues to climb [27], and the Centers for Disease Control has predicted that thyroid cancer will be among the four cancers with the largest increases in women by 2020 [28, 29]. Thyroid cancer mortality also appears to be increasing in the United States [30–32] in contrast to global trends which show declining thyroid cancer mortality for males and females in most other countries [32]. Thyroid cancer also impacts quality of life and is the most likely of all cancers to lead to personal bankruptcy in the first year following diagnosis due to ongoing treatments and side effects of treatment [33]. While a part of the rapid rise in incidence of thyroid cancer appears attributable to increased diagnostic technology and screening, recent reports suggest a true increase in incidence of all stages of thyroid cancer and aggressive disease for which environmental risk factors may play a role [30, 31, 34]. Identifying environmental risk factors would aid efforts towards disease reduction and prevention of aggressive disease in particular [35]. Given the paucity of sufficiently powered studies to investigate the incidence of thyroid cancer in airline crew, we conducted a first-step meta-analysis of cohort studies that reported standardized incidence ratios (SIR) and standardized mortality ratios (SMR) of thyroid carcinoma in cockpit and cabin crew.

## Methods

This study was carried out following the Preferred Reporting Items for Systematic Reviews and Meta-analyses guidelines [36].

### Search strategy

Relevant studies were identified by searching PubMed and Cochrane Database and scanning reference lists of articles. The following search terms were used: (Flight crew* OR Flight attendant* OR Air*crew* OR Airline* OR Airplane* OR Flight* OR Pilots* OR Steward* OR Stewardess OR Cabin crew* OR Cabin attendant* OR Aviat*) AND cancer AND (incidence OR mortality OR risk OR probability OR death OR health OR prevalence). The date of the last search on PubMed and Cochrane Database was March 13, 2017. We also evaluated related review articles to identify studies that were missed by searching PubMed and Cochrane Database.

### Inclusion criteria and study selection

Studies that reported a SIR or a SMR for thyroid or endocrine cancer in cockpit or cabin crew or that reported observed and expected counts that could be used to calculate a SIR or SMR were considered eligible for inclusion. Some studies reported a SIR or SMR of “thyroid and other endocrine cancers” or “endocrine cancers”. We included these studies because thyroid cancer is the most common endocrine malignancy, and constitutes the majority of new diagnoses and deaths among endocrine malignancies each year [37]. We also repeated the meta-analysis without these studies to confirm that their inclusion did not significantly change the study results.

Two authors (A.C. and M.R.) assessed the eligibility of studies, with any disagreements settled by consensus with a third author (G.S.L.). Initial screening was done using the article title and abstract to generate a list of potentially relevant studies. Each study in the list then underwent full text review to determine eligibility by our inclusion criteria. If different publications reported on subsets of duplicative data, we included the study with the largest amount of data, e.g. the study that followed the same cohort for the longest period of time.

### Data extraction

For each included study, we extracted data available about the study participants’ characteristics (e.g. gender, nationality, and occupation); data collection period; person-years of follow up; outcomes; and whether the outcomes were attributed to thyroid cancer specifically, thyroid and other endocrine cancers, or endocrine cancers in general (unspecified). Mean follow up was calculated by dividing person-years of follow up by the number of participants.

### Statistical analysis

We calculated the summary SIR and SMR for all airline crew, and also calculated these values for airline crew stratified by location on the plane. The two location groups were cockpit crew (e.g. pilots) and cabin crew (e.g. flight attendants).

We used Stata version 13.1 (StatCorp) to perform random effects model and fixed effects model meta-analyses, producing summary relative risks and 95% confidence intervals. Unless otherwise specified, reported SIR and SMR results were obtained using the random effects model. All statistical tests were 2-sided. The I-squared measure of heterogeneity [38] was considered significant at *p* = 0.10. Funnel plots were created using Stata to assess for potential publication bias across studies. To assess the goodness of fit of linear regressions, the Pearson correlation coefficient *R*^2^ was calculated using Microsoft Excel 2013.

## Results

**Fig. 1:**
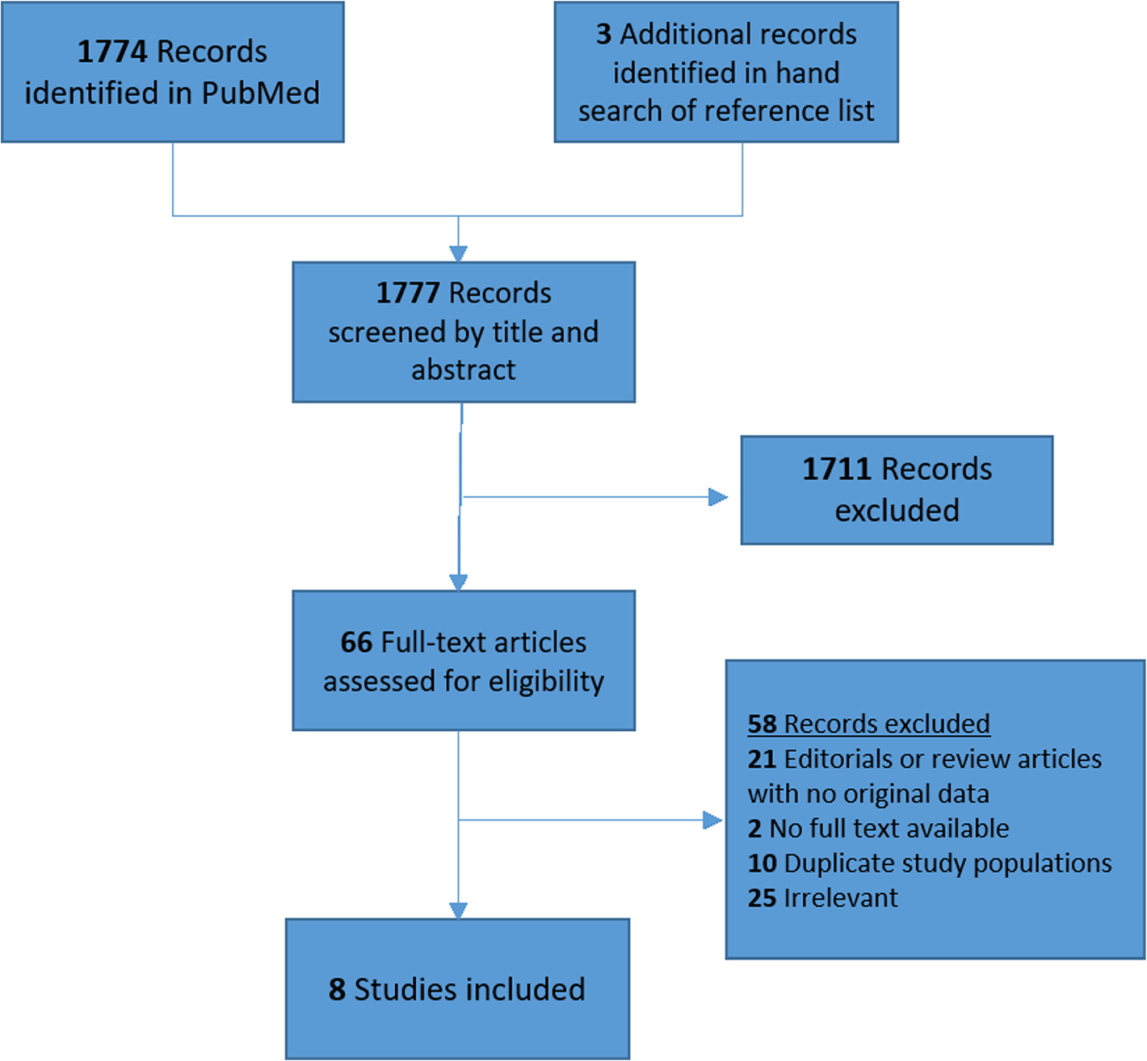
Preferred reporting items for systematic reviews and meta-analysis flowchart of article search in PubMed and study selection. Eight studies met inclusion criteria and were included in the meta-analysis

Our search terms on PubMed produced 1774 results. Our search terms on Cochrane Database produced two systematic reviews and 26 controlled trials, all of which were excluded on initial screening. We also searched reference lists and review articles to identify an additional three publications [39–41]. We performed initial screening of the 1777 records on PubMed by titles and abstracts, yielding 66 records that were assessed by full text review for eligibility. Among the 66 records that underwent full text review, eight studies met inclusion criteria and were included (Fig. 1). Ten publications [42–51] were excluded due to overlapping cohorts with later follow-up studies. Six of these publications [42–44, 46, 47, 51] provided information on cohort members documented in a later and more inclusive publication by Hammer et al. [52], and four publications [45, 48–50] were more inclusively documented by Pukkala et al. [53]. Additionally, two studies were excluded because their full texts were not available [54, 55].

This review included 8 studies published between 1996 and 2014, incorporated data collected between 1943 and 2008 from 11 countries (Denmark, Finland, Germany, Greece, Iceland, Italy, Norway, Sweden, UK, USA, and Canada), and included 243,088 airline crew members (Table 1). Five of the included studies reported data on cockpit crew only [39, 40, 56–58], two reported on cabin crew only [53, 59], and one reported on cockpit and cabin crew [52].

**Table 1:** Studies included in the meta-analysis

Source	Data Collection	Country	Population	Total No. (M/F)	Person-years	Thyroid cancer SIR (95% CI)	Thyroid cancer SMR (95% CI)
Pukkala et al., 2002 [56]	Various years by country (1943 to 1996 in Denmark, 1953 to 1997 in Finland, 1984 to 1997 in Iceland, 1962 to 1996 in Norway, and 1961 to 1996 in Sweden)	Scandinavia (Denmark, Finland, Iceland, Norway, Sweden)	Cockpit crew	10,032 (10,032/0)	177,244	0.88 (0.18–2.58)	–
Reynolds et al., 2002 [59]	1988–1995	US	Cabin crew	52,741 (8,720/ 44,021)	Not stated	0.25 (0–1.43) female only	–
Yong et al., 2014 [57]	1960–2008	US	Cockpit crew	5,964 (5,958/6)	202,316	–	0.77^a^(0.09–2.79)
Hammer et al., 2014 [52]	1989–2004	Denmark, Finland, Germany, Greece, Iceland, Italy, Norway, Sweden, UK and the USA	Cockpit crew	36,816 (36,816/0)	747,123	–	1.06^a^(0.28–2.72)
			Cabin crew	44,667 (0/44,667)	1,033,146	–	1.53^a^(0.47–3.69)
dos Santos Silva et al., 2013 [58]	1989–2008	UK	Cockpit crew	16,329 (15,867/462)	285,259	0.35^a^ (0.04–1.26)	–
Pukkala et al., 2012 [53]	1947–2005	Finland, Iceland, Norway, and Sweden	Cabin crew	10,066 (8,507/1,559)	237,627	1.01 (0.58–1.61)	–
Grayson et al., 1996 [39]	1975–1989	US Air Force	Cockpit crew	59,940 (59,940/0)	532,980.97	1.61^b^(0.81–2.83; 99% CI)	–
Nicholas et al., 2001 [40]	1970–1998	US and Canada	Cockpit crew	6,533 (6,533/0)	118,418	1.08 (0.40–2.89)	–

^a^thyroid and other endocrine

^b^endocrine

**Fig. 2:**
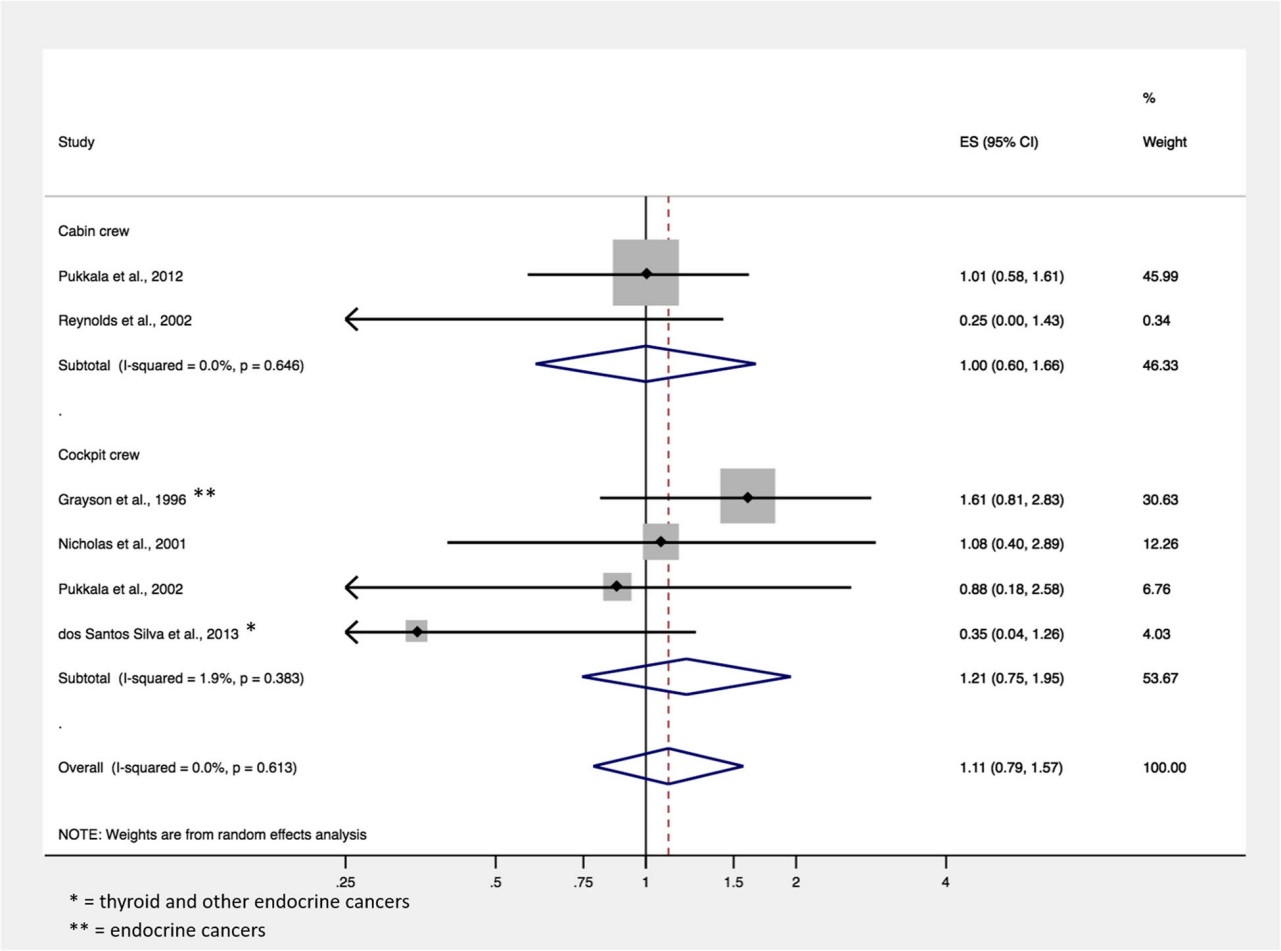
Standardized incidence ratio of thyroid cancer in the studies included in the meta-analysis. Unless otherwise specified, SIR data is reported for thyroid cancer, specifically

The overall summary SIR of thyroid cancer in individuals of any aircrew-based occupation (cockpit and cabin) was 1.11 (95% CI, 0.79–1.57; *p* = 0.613; 6 records). The summary SIR for cockpit crew was 1.21 (95% CI, 0.75–1.95; *p* = 0.383; 4 records) and the summary SIR for cabin crew was 1.00 (95% CI, 0.60–1.66; *p* = 0.646; 2 records) (Fig. 2). Excluding data that was not specific to thyroid cancer (i.e. data reported for “thyroid and other endocrine cancers” or “endocrine cancers”) did not significantly change the overall summary SIR.

**Fig. 3:**
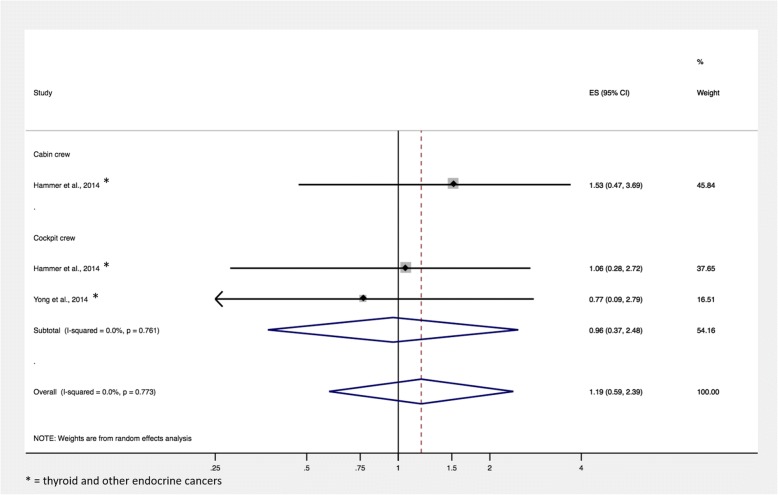
Standardized mortality ratio of thyroid cancer in the studies included in the meta-analysis. Unless otherwise specified, SMR data is reported for thyroid cancer, specifically

The overall summary SMR for thyroid cancer in airline crew was 1.19 (95% CI, 0.59–2.39; *p* = 0.773; 2 records). The summary SMR for cockpit crew was 0.96 (95% CI, 0.37–2.48; *p* = 0.761; 2 records) (Fig. 3). The summary SMR for cabin crew was not calculated because only one record reported SMR data for cabin crew, which was 1.53 (95% CI, 0.47–3.69; 1 record) [52]. The overall summary SMR of thyroid cancer specifically (excluding data reported for “thyroid and other endocrine cancers” or “endocrine cancers”) was not calculated because no record reported SMR data for thyroid cancer specifically.

In addition to the above results obtained using the random effects model, no significant increase in SIR or SMR was observed using the fixed effects model. Funnel plots, despite the small number of studies that may inhibit a full definitive evaluation, indicated there were no obvious outliers and no direct evidence of publication bias (data not shown).

Thyroid cancer is indolent relative to breast cancer, leukemia, and melanoma [60]. Presentation of thyroid cancer may differ from other cancers, in which case follow-up time could be a limiting factor. To address this issue, we assessed whether studies with longer follow-up tended to report higher thyroid cancer SIR. For the five studies that reported thyroid cancer SIR and follow-up data [39, 40, 53, 56, 58], we observed no significant association between SIR and mean follow-up time (Pearson correlation coefficient: *R*^2^ = 0.29, *p* = 0.31). There was also no significant association between SIR and total person-years of follow-up (Pearson correlation coefficient: *R*^2^ = 0.27, *p* = 0.34) or duration of data collection (Pearson correlation coefficient: *R*^2^ = 0.06, *p* = 0.62). These statistical analyses are presented in Additional file 1.

## Discussion

We conducted, to our knowledge, the first meta-analysis to investigate the risk of thyroid cancer in airline cockpit and cabin crew members. We found no significant increase in incidence of or mortality due to thyroid cancer relative to the general population. However, the measurement of accumulated radiation exposure in flight is imprecise and childhood exposure windows are not captured in the studies included in this meta-analysis. Assigning equal exposures to all airline crew could mask a threshold effect in which only airline crew members with the highest radiation exposure levels or some lifetime combination of childhood and adult radiation exposure levels experience increased thyroid cancer risk. It is also possible that, in contrast to findings that childhood radiation exposure is clearly associated with thyroid cancer risk later in life, when the thyroid is exposed to radiation during adulthood [21, 61] the impacts may not be as evident. Therefore, our results may be consistent with the notion that exposure to radiation in adulthood does not predispose to a large increase in the risk of thyroid cancer.

We were unable to assess risk by gender given the data provided in the publications. Gender is an important factor given the three-fold higher incidence of thyroid cancer in women compared with that in men in the general U.S. population [27] and documentation that thyroid cancer is particularly elevated among women of reproductive age with incidence rates that are second only to breast cancer [62]. For similar reasons, we were unable to assess risk considering cigarette smoke exposure for airline crew working prior to 1990. If the included studies were clinical trials designed and randomized to eliminate smoking as a confounder, then it might be feasible to conduct a sensitivity analysis. Similar to gender, age also plays a role in the incidence of thyroid cancer [63] and should be delineated in further studies with patient level data. We encourage next-step work to consider industry participation in order to better capture specific variables such as gender, age, and primary and secondary smoking data for airline crew.

Our results may reflect the inability to consider these specific variables and a small number of studies with insufficient person-years of follow-up to detect small to moderate increases in risk. Unlike the studies for melanoma and breast cancer for example, the number of cases of thyroid cancer remain small in comparison requiring larger datasets with extensive person-years of follow-up. Identifying more studies or including additional person-years of follow-up in existing studies would produce narrower confidence intervals of outcome estimates to detect small increases in risk. In order to assess small or moderate risks of radiation-associated thyroid cancer in airline crew, future meta-analyses would need to include more studies than the number included here, especially if the studies included were of low or moderate power and hindered by lack of detail on other key variables.

This study has several other limitations. To more thoroughly understand the risk of radiation exposure among adults, lifetime individual radiation exposure levels should be captured based upon childhood exposures and adult occupation-based exposures, perhaps by using innovative means for analyzing flight frequency, duration, and elevation data that would require industry participation. Changes in screening, incidence, and mortality of thyroid cancer over different time periods, as well as clinical practice differences in countries outside the United States, were not adjusted for when calculating the SIR and SMR. We considered multiple hypothesis testing from including and excluding other endocrine cancers, and regard it as unlikely to have affected our conclusions given the insignificant results. Our meta-analysis of thyroid cancer mortality is also limited because the published studies reported SMR data for thyroid cancer that included other endocrine malignancies.

There are a number of strengths of this study. One is that significant heterogeneity was not observed in the overall SIR and SMR (I-squared = 0.0%, *p* > 0.10). Another strength is that participants were stratified by airline crew occupation (i.e. cockpit or cabin crew) to assess whether location on the airplane was a confounding factor. We did not find a significant difference in thyroid cancer SIR or SMR between the two airline crew occupation groups, suggesting that location on the airplane was not a confounding factor. We also addressed the potential limitation of insufficient follow-up, given that indolent thyroid cancers may present later and require a greater duration to effectively monitor. We did not find evidence supporting a trend between mean follow-up and reported SIR across studies, suggesting that insufficient follow-up is less likely to be a concerning factor in the analysis.

Understanding whether radiation exposure during airplane flights is an environmental risk factor for thyroid cancer in men and women is important given the increased use of air travel by the public. Moreover, if airline crew represent the group with the highest exposure levels yet have gender-specific risks similar to the general population, this suggests resources should be directed to evaluate other environmental factors given increasing thyroid cancer rates. The study of occupational cohorts can be critical to inform understanding of environmental risk factors driving the rise in thyroid cancer incidence in recent decades [27, 30]. Future work should contemplate improved exposure assessments and a larger dataset that includes specific variables.

## Conclusions

Airline crew were not found to have a significantly elevated risk of thyroid cancer incidence or mortality relative to the general population. Future research should capitalize on the growing occupational cohort dataset and employ innovative methods to quantify lifetime radiation exposure to further assess thyroid cancer risk in airline crew.

## Additional file

Additional file 1: Statistical analysis to assess association between thyroid cancer SIR and follow-up data. (XLSX 98 kb)

## Abbreviations

SIR: Standardized incidence ratio
SMR: Standardized mortality ratio
